# Factors affecting relapse in patients with Granulomatosis Polyangiitis: a single-center retrospective cohort study

**DOI:** 10.3906/sag-2008-217

**Published:** 2021-08-30

**Authors:** Müge AYDIN TUFAN, Nihan TEKKARIŞMAZ, Ahmet Eftal YÜCEL

**Affiliations:** 1 Department of Rheumatology, Faculty of Medicine, Başkent University, Ankara Turkey; 2 Department of Nephrology, Başkent University Adana Dr Turgut Noyan Research and Medical Hospital, Adana Turkey

**Keywords:** Granulomatous polyangiitis, relapse, relapse

## Abstract

**Background and aim:**

This study aimed to determine the frequency of relapse, the risk factors for relapse, and the correlation of relapse with immunosuppressive regimens in patients with granulomatosis polyangiitis (GPA).

**Materials and methods:**

The demographic characteristics, the clinical, laboratory, and radiological findings, the immunosuppressive treatment regimens, and the remission and relapse rates of 50 patients with GPA were obtained retrospectively from medical records.

**Results:**

: The mean relapse-free survival rates at years 1, 3, and 5 were 82%, 60%, and 50%, respectively. Increased relapse rates were observed in patients who had cavitary lung lesions (52.2% vs. 22.2%, p = 0.04) and in those who had elevated serum creatinine levels (1.8 vs. 0.9, p = 0.00). The patients received two different types of remission induction therapies; 36% of them received the combination therapy involving cyclophosphamide (CYC) and rituximab (RTX), and 62% received CYC alone. Relapse was observed in 22.3% of the patients who received the combination remission induction therapy and in 61.3% of the patients who received CYC alone (P = 0.003).

**Conclusion:**

An increased risk of relapse was observed in patients who had cavitary lung lesions and in those who had elevated serum creatinine levels. The combined use of RTX and CYC for the remission therapy in GPA reduced the relapse rates compared with the use of CYC alone

## 1. Introduction

Granulomatosis polyangiitis (GPA) is a form of necrotizing granulomatous vasculitis associated with antineutrophil cytoplasmic antibodies (ANCA), and it affects small- and medium sized blood vessels [1]. GPA is diagnosed based on clinical, serological (ANCA positivity), and histopathological findings. While GPA affects a wide array of organs, it is most frequently encountered in the respiratory tract and in the kidneys [1]. GPA is a severe condition that can manifest as rapid organ damage and can lead to death if left untreated. The 5-year survival rate for GPA has risen by up to 70%–78% as a result of the use of novel immunosuppressants and biologic agents [2,3], and its 5-year relapse rate is approximately 40%–50% [1,4]. 

The primary goal of GPA treatment is to achieve remission, and the secondary goal is to prevent relapse (maintenance) [5]. Relapse in GPA is especially concerning due to aggravated organ damage that can be life-threatening. To avoid relapse, clinicians prefer administering intensive and long-term immunosuppressive treatments. While these treatments prevent relapse in half of GPA patients, the long-term use of immunosuppressants at high doses involves other risks secondary to their cumulative side effects (e.g., secondary malignancy, infection, and increased mortality) [5]. Yet, a consensus on how to reduce relapse and when to stop immunosuppressive therapy in patients with GPA remains nonexistent. Thus, this study aimed to determine the frequency and risk factors of relapse in GPA patients.

## 2. Materials and methods

### 2.1. Participants

This study was approved by the Başkent University Institutional Review Board (project no.: KA19/287) and was supported by the Başkent University Research Fund. Fifty adult patients who were diagnosed with GPA and were treated at the Rheumatology Department of Başkent University Adana Dr Turgut Noyan Research and Medical Hospital between January 2005 and August 2019 were included in this study. GPA diagnosis was based on the American College of Rheumatology 1990 criteria. Data including demographic characteristics, clinical, laboratory, and radiological findings, organ involvement status, and Birmingham vascular activity score (BVAS) were obtained retrospectively from the patients’ medical records. Erythrocyte sedimentation rate (ESR), C-reactive protein (CRP), serum creatinine level, hemoglobin level, cANCA and pANCA serology (measured with the enzyme-linked immunosorbent assay method), and presence of proteinuria and hematuria at the time of diagnosis were all noted. Lung findings (presence of ground-glass opacity, cavity, nodule, and/or bronchiectasis) obtained by chest X-rays and thoracic computed tomography were also noted. The immunosuppressants (cyclophosphamide (CYC), rituximab (RTX), azathioprine (AZA), methotrexate (MTX), mycophenolate mofetil (MMF), and prednisolone) administered for the remission induction and maintenance therapies, the duration of treatment, and the information concerning plasmapheresis were noted. Additionally, complications such as life-threatening infections and the development of end-stage renal disease that required dialysis were recorded. Data on remissions, relapses, and mortality for all patients were also collected. 

### 2.2. Clinical procedure

CYC and corticosteroid were administered in the remission induction therapy. CYC (500 mg) was administered intermittently (every 10 days during the 1st month, every 2 weeks during the next 2 months, and then every 6–8 weeks depending on the disease activity) and intravenously. Prednisolone was administered intravenously at 500 mg/day for 3–5 days depending on the disease activity. For the maintenance therapy, peroral prednisolone was administered at 1 mg/kg/day. The prednisolone dose was tapered such that the 10–15 mg/day dose was achieved within 3 months. All patients received the same dose of prednisolone at the beginning of the treatment; in addition, patients received a similar reduction regimen. After 2011 (upon receiving the approval from the Turkish Ministry of Health), RTX was administered twice with a 2-week interval at a total dose of 2 g (repeated every 6 months) for the GPA patients who relapsed or who had a refractory disease. RTX alone was not used in the remission induction therapy. Patients with severe kidney damage and/or alveolar hemorrhages underwent 5–7 cycles of plasmapheresis. For the maintenance therapy, AZA (at a dose of 2 mg/kg/day during the first 12 months, 1.5 mg/kg/day between months 12–18, and 1 mg/kg/day after month 18), MTX (at a dose of 15–20 mg/week), MMF (2 g/day), or RTX were administered. 

For the treatment of relapse, the dose of the currently administered corticosteroid in the maintenance therapy was increased, the frequency of intravenous CYC administration was increased, or RTX was administered. All patients who were administered with CYC and RTX also received per oral trimethoprim/sulfamethoxazole (800/160 mg) 3 days a week for 6 months for *Pneumocystis jirovecii* prophylaxis during the induction.

### 2.3. Statistical analysis

The SPSS 25.0 software package (IBM Corp., Armonk, NY, USA) was used in the statistical analysis of the data. Categorical measurements were expressed in numbers and percentages and continuous measurements were expressed in mean and standard deviation (median and minimum-maximum, where necessary) values. The chi-square test or Fisher’s test was used in the comparison of categorical variables. Distributions were analyzed for a comparison of continuous measurements between the groups, wherein the Student’s t-test was used for variables with a parametric distribution and the Mann–Whitney U test for variables with a nonparametric distribution. Survival curves for relapse and mortality were created using the Kaplan–Meier analysis. A value of p < 0.05 was considered statistically significant in all tests.

## 3. Results

### 3.1. Patients’ profile

This study involved 50 patients whose mean age was 52.8 ±15.8 years, and 58% of them were female. The mean follow-up period was 47 (3–180) months. Of the patients, 68% and 26% tested positive for cANCA and pANCA, respectively. Diagnoses were confirmed with tissue biopsies in 76% of the patients. The patients’ baseline demographic, clinical, and laboratory data are shown in Table 1.

**Table 1:** Patients’ baseline demographic, laboratory and clinical data.

Parameters	Values
Sex (female)*	29 (58)
Age (years)**	52.8 ± 15.8
Follow-up duration (months)***	47 (3–180)
Survival (months)**	139.2 ± 11.9
Laboratory data	
ESR, mm/h**	74.6 ± 27.7
C-Reactive protein, mg/dL***	93 (9–220)
Creatinine, mg/dL***	1.3 (0.5–13.8)
Hemoglobin, g/dL**	10.6 ± 1.9
Proteinuria, g/day***	1.5 (0–9)
PR3-ANCA positivity*	34 (68)
MPO-ANCA positivity*	13 (26)
BVAS score**	20.2 ± 6.4
Clinical data	
Constitutional symptoms*	46 (92)
Upper respiratory tract involvement*	35 (70)
Ear involvement*	16 (32)
Lung involvement*1. Ground glass opacities*2. Nodular lesions*3. Alveolar hemorrhage*4. Cavitary lesions*5. Bronchiectasis*	43 (86)25 (50)27 (54)19 (38)18 (36)6 (12)
Kidney involvement*1. Proteinuria*2. Hematuria*3. Proteinuria and hematuria* 4. Renal failure*	36 (72) 34 (68)33 (66) 32 (64) 14 (28)
Eye involvement*	6 (12)
Musculoskeletal system involvement*	23 (46)
Skin involvement*	5 (10)
Nervous system involvement*	5 (10)
Gastrointestinal tract involvement*	5 (10)
Cardiovascular system involvement*	1 (2)
Lung and kidney involvement*	22 (44)

*n (%), **mean ± SD, ***median (min–max).

### 3.2. Baseline clinical results

Nearly all (92%) patients presented with symptoms, such as fatigue, weight loss, and fever. Involvement was most frequently observed in the lungs (86%), kidneys (72%), and upper respiratory tract (70%). Moreover, 44% of the patients (n = 22) had both renal and pulmonary involvement (Table 1).

The remission rate was 80%, and the rate of refractory disease was 20%. Additionally, 12% of the patients exhibited full remission, and immunosuppressive therapy was discontinued in these patients. Relapse was observed in 46% of the GPA patients. The remission rates on the 6th, 12th, and 15th month were 16% (8 patients), 52% (26 patients), and 76% (38 patients), respectively.

For the induction therapy, in addition to the intravenous steroid therapy, CYC alone was administered in 62% of the patients and both CYC and RTX were administered in 36% of the patients. For the maintenance therapy, the patients were administered with AZA (66%), RTX (46%), MTX (22%), and MMF (2%). The median treatment duration was 36 (min 0, max 120) months for corticosteroids and 24 (min 1, max 48) months for AZA. The doses for corticosteroids were tapered and discontinued in 38% of the patients. The median total amount of CYC and RTX administered per patient was 6 g (1.5–13.5 g) and 4 g (2–14 g), respectively.

In total, 12 GPA patients (24%), including 3 patients with renal involvement and 9 patients with renal and pulmonary involvement, underwent plasmapheresis.

### 3.3. Clinical results related to relapse

The relapse rate was higher in patients with cavitary lung lesions than in those without cavitary lung lesions (52.2% vs. 22.2%, p = 0.04). The relapse rate was also higher in patients with high creatine levels than in patients with low creatine levels (1.8 vs. 0.9, p = 0.00). The mortality rates were 30.4% and 7.7% in patients with and without a history of relapse, respectively (P = 0.06) (Table 2). A multivariate logistic regression analysis was performed for variables that were significant in the univariate analyses. However, an independent risk factor affecting relapse was not detected.

**Table 2:** Comparison of relapsing and non-relapsing patients.

	Relapsing group (n = 23)	Nonrelapsing group (n = 27)	p
Age (years)**	54.4 ± 15.2	51.4 ± 16.4	0.59
Sex (female)*	11 (47.8)	18 (66.7)	0.25
Renal involvement*	18 (78.3)	18 (66.7)	0.52
Lung involvement* Ground glass opacities* Nodular lesions* Alveolar hemorrhage* Cavitary lesions* Bronchiectasis*	20 (87)12 (52.2)12 (52.2)10 (43.5)12 (52.2)3 (13.0)	23 (85.2)13 (48.1)15 (55.6)9 (33.3)6 (22.2)3 (11.1)	1.001.001.000.560.041.00
Ears involvement*	4 (17.4)	12 (44.4)	0.06
Upper respiratory tract*	18 (63.0)	17 (63.0)	0.35
Eye involvement*	4 (17.4)	12 (44.4)	0.06
Musculoskeletal involvement*	11(47.8)	12 (44.4)	1.00
Skin involvement*	1 (4.3)	4 (14.8)	0.35
PR3-ANCA positivity*	14 (60.9)	20 (74.1)	0.37
MPO-ANCA positivity*	8 (34.8)	5 (18.5)	0.21
Dialysis, at the time of diagnosis*	9 (39.1)	5 (18.5)	0.12
Serious infection*	9 (39.1)	4 (14.8)	0.06
Mortality*	7 (30.4)	2 (7.7)	0.06
Corticosteroids duration (months)**	60 ± 42	38 ± 30	0.06
ESR, mm/h**	77 ± 29	72 ± 26	0.55
CRP, mg/dL**	103 ± 72	88 ± 55	0.51
Serum creatinine, mg/dL***	1.8 (0.7–13)	0.9 (0.5–9.3)	0.00
Hematuria*	18 (78.3)	15 (55.6)	0.13
Proteinuria, g/day***	1.7 (0.0–9.0)	1.1 (0.0–5.0)	0.22
BVAS**	21.6 ± 6.8	18.9 ± 5.8	0.08

*n (%), **mean ± SD, ***median (min–max), p < 0.05 significant.

Age, sex, hemoglobin, sedimentation, CRP, ANCA serology, BVAS, and duration of steroid therapy were not found to be risk factors for relapse. Similarly, skin, kidney (hematuria and proteinuria), lung (ground-glass infiltration, alveolar hemorrhage, pulmonary nodules, and bronchiectasis), upper respiratory tract (otitis and sinusitis), musculoskeletal system, eye, heart, GI tract, and neurological system involvement were not determined as risk factors for relapse (Table 2).

### 3.4. Induction therapy

The combined use of RTX and CYC in remission induction therapy was applied in 36% of the patients (n = 18). Of these patients, 77.7% (n = 14) achieved remission and 22.3% (n = 4) suffered a relapse (p = 0.025)

In the remission induction therapy, the monotherapy involving CYC was applied in 62% of the patients (n = 31). Of these patients, 38.7% (n = 12) achieved remission and 61.3% (n = 19) suffered a relapse (p = 0.001). 

The frequency of relapse was found to be significantly lower in patients who received the combination therapy than in patients who received the monotherapy (p = 0.003). 

### 3.5. Maintenance therapy

In the maintenance therapy, no difference in terms of relapse was observed between patients who received RTX and those who received AZA (60.8% vs. 80%, p = 0.520), between patients who received RTX and those who received MTX (60.8% vs. 34.8%, p = 0.769), and between patients who received AZA and those who received MTX (80% vs. 4.8%, p = 0.294). Thus, in the maintenance therapy, no significant differences were observed among the AZA, MTX, and RTX therapy in terms of relapse rates.

### 3.6. Relapse-free survival rates

The mean relapse-free survival rates of the patients at years 1, 3, and 5 were 82%, 60%, and 50%, respectively. The estimated mean relapse-free survival period was 77.1 ± 13.3 (95% CI: 51–103) months (Table 3 and Figure).

**Table 3:** Relapse-free survival rates of patients with GPA.

	Estimatemeana	Std. error	95% confidence interval		1-year survivor %	3-year survivor %	5-year survivor %	9-year survivor %
			Lower bound	Upper bound				
Relapse-free survival (months)	77.1	13.3	51.0	103.1	82.6	60.7	50	25

**Figure:**
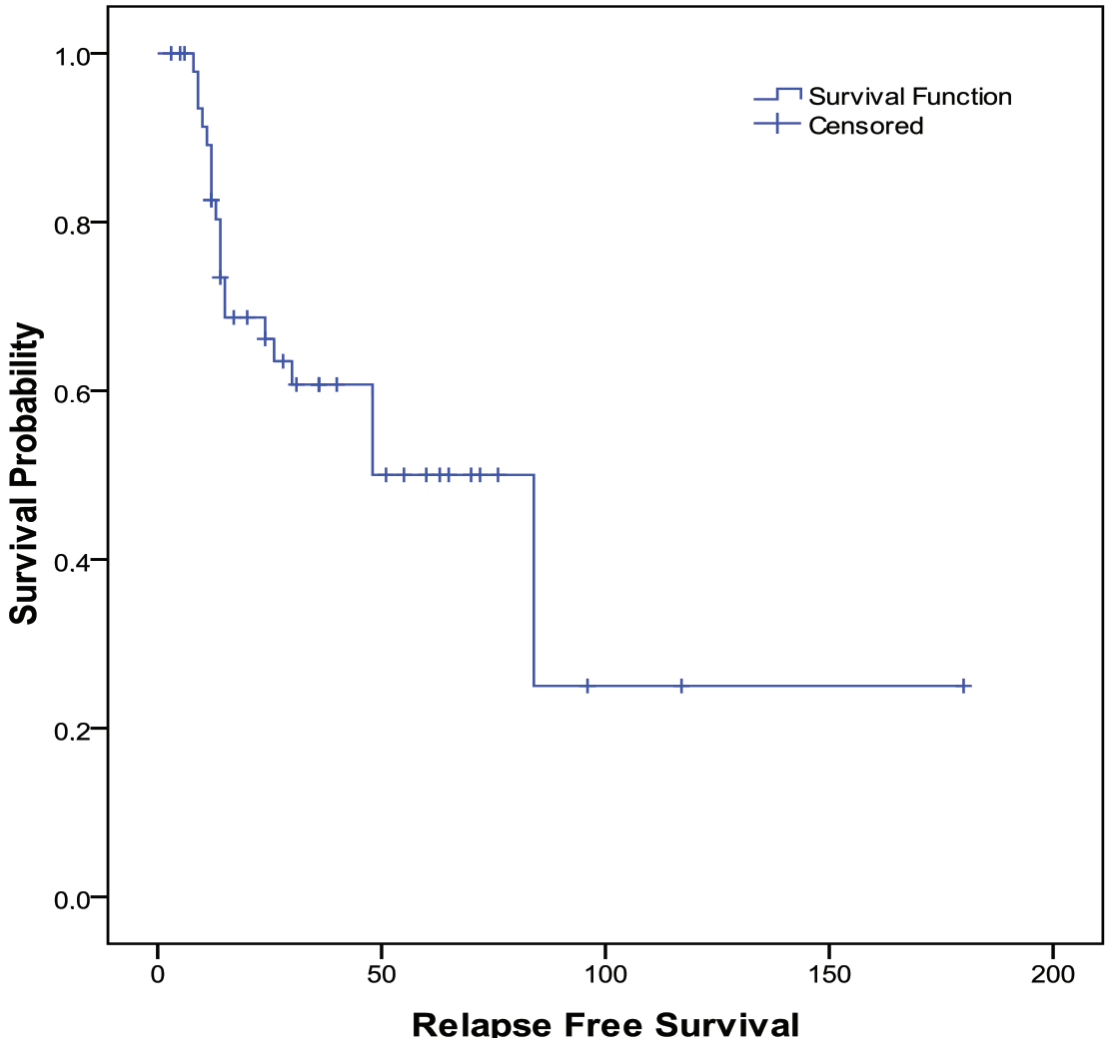
Kaplan–Meier curve of relapse-free survival (months).

Of the patients, 28% required hemodialysis at the time of diagnosis, 18% required hemodialysis after the treatment, and 26% experienced a life-threatening infection.

## 4. Discussion 

### 4.1. Risk factors influencing the relapse rate in GPA

This study investigated the relapse rates and the risk factors affecting relapse in 50 patients who were diagnosed with GPA and treated in a single center. Although a marked improvement was achieved in terms of mortality and progression to end-stage renal disease in GPA patients in the preceding years, the GPA relapse rates remain high [4]. 

The clinical and demographic data of our patients were similar to those reported in previous studies [6,7]. However, the lung involvement rate was higher in our patients than that reported in the literature (86% vs. 62%–67%) [6,7]. Similarly, the rate of pulmonary–renal involvement was higher in our study than in previous reports (44% vs. 14.7%) [6]. This finding indicates that the present cohort experienced more major organ involvements, such as the lung and the kidney. The refractory disease rate (20% vs. 23%) and relapse rate (46% vs. 42%) in our study were similar to those reported by Hogan et al. [8]. Moreover, the reported 5-year overall relapse rate in GPA patients was 50% [5,9,10], and a similar rate (50%) was found in our study. 

Pagnoux et al. found that lung involvement is a predictor of relapse [4]. Another study also reported that lung involvement was a risk factor for relapses [8]. By contrast, our study found that lung involvement is not a risk factor for relapse. We investigated the lung involvement subtypes, namely, bronchiectasis, and solid nodules, and we found that they are not risk factors for relapse. By contrast, Lhote et al. [11] reported that relapses were more common in patients with bronchiectasis in ANCA-associated vasculitis.

Whether the presence of cavitary lung lesions at the time of GPA diagnosis is a risk factor is controversial. In our study, the patients with cavitary lung lesions had higher relapse rates than those without cavitary lung lesions (52.2% vs. 22.2%, P = 0.04). Russell et al. [12] also reported that relapse was more common among patients who had cavitary lung lesions. By contrast, Komocsi et al. reported that the presence of cavitary lesions and solid nodules in the lungs are not risk factors for GPA relapse [13].

The upper respiratory tract involvement is also a risk factor for relapses in GPA as reported by Hogan et al. [8]. However, we found that upper respiratory tract involvement was not a risk factor for relapse.

The rate of relapse involving the same organ as that involved at the time of diagnosis was reported to be 70.9% [14], whereas in our study, the rate was 78.2%. The rate of relapse involving a new organ (de novo) was reported to be 46% [7], whereas it was found to be as low as 21.8% in our patient series.

A review of four clinical trials reported that cardiovascular involvement is a risk factor for relapse [15]. Unfortunately, this finding could not be verified given that only one patient in our study had cardiac involvement. 

Furthermore, low serum creatinine levels at the time of diagnosis were reported to be associated with increased risk of relapse rates in GPA patients [3,7,15,16] wherein the serum creatinine cut-off value was 1.13 mg/dL (100 mmol/L) or 2.26 mg/dL (200 mmol/L). We also observed an increased risk of relapse in patients with elevated creatinine levels. Creatinine was associated with relapses at a cut-off value of 1.15 mg/dL with 78.3% sensitivity, 60% specificity, and 69.9% accuracy (95% CI: 0.55–0.84). This finding, however, differed from previous findings due to the following reasons: (1) our patient group consisted only of patients with active GPA and (2) immunosuppressants were preferred at a lower cumulative dose in patients with renal involvement in our study to avoid the increased risk of drug toxicity and secondary infections. 

The presence of persistent hematuria has been reported to increase the risk of relapse [17], contrary to our findings where hematuria is not a risk factor for relapse.

Roderau et al. [7] reported that relapse frequency was lower in patients with skin involvement. In another study, the presence of cutaneous ischemia, which indicates skin involvement, has been shown to increase the risk of relapse [18]. By contrast, only five of our patients displayed skin involvement, indicating that skin involvement is not a risk factor for relapse.

Some studies reported that a positive ANCA test is a risk factor for relapse, but this report is contrary to our findings [3]. Tomasson et al. [19] suggested that ANCA titers should be monitored in GPA patients who are in remission because a persistent ANCA titer positivity or a new positivity during the follow-up could indicate a possible future relapse. In the current study, all patients who stopped receiving treatment due to full remission remained ANCA negative. We conclude that the changes in ANCA test results during follow-up should be carefully monitored, and this approach could offer guidance in reducing the dose of immunosuppressive agents.

### 4.2. Effects of different immunosuppressive regimens on relapse in GPA

#### 4.2.1. Remission induction

CYC and corticosteroid therapies are the cornerstone of remission induction in the treatment of ANCA-associated vasculitis [20]. In recent years, RTX was shown to be as effective as CYC in both induction and maintenance therapies [20]. The rate of CYC and corticosteroid usage in remission induction is 74%–78% [6,21]. In our study, CYC and corticosteroid were used in 98% of the patients for the remission induction therapy. The higher rate of CYC and corticosteroid usage in our series is attributed to the high number of patients with vital organ involvement.

The rate of RTX usage in remission induction therapy was 7.1% as reported in the Polvas registry [21]. In another study, the rate of RTX usage in induction was 5.8% in patients in whom CYC use was ineffective [6]. In the current study, the rate of use of the RTX + CYC combination in remission induction therapy was 36%.

In the RITUXVAS study and in our study, the GPA patients were administered with the RTX + CYC combination therapy for remission induction [22]. In the RITUXVAS study, no significant differences in terms of remission, relapse, and mortality were observed between the groups that were separately administered with CYC and CYC + RTX [22]. 

In the current study, relapse was less common in the group that was administered with pulse CYC+RTX for remission induction than in the group that was administered with CYC alone (22.3% vs. 61.3%, P = 0.003). In our opinion, this data is important. Further prospective clinical studies involving larger patient populations should be conducted in order to determine the effects of RTX use for induction on the frequency of a relapse.

A multicenter study [20] compared the use of RTX and CYC in remission induction therapy. The remission rate was 67% in the RTX group and 42% in the CYC group. RTX was found to be as effective as CYC in cases involving major kidney disease and alveolar hemorrhage [20]. 

The mean total dose of CYC administered in previous studies was 7.9–14 g [21,23], whereas that in our study was lower. As for RTX, the mean total dose administered in previous studies was 2–8 g [21,23], which is similar to that used in our study.

Cortazar’s study showed that combination therapy with rituximab and cyclophosphamide is highly efficacious, allows for rapid tapering of high-dose glucocorticoids [24]. Pepper et al. suggested that rapid withdrawal of corticosteroids within 2 weeks is feasible with the RTX/CYC combined remission induction regimen [25]. Similarly, in our study, the median total amount of CYC administered per patient was 6 g (1.5–13.5 g). Moreover, RTX was not added to the treatment regimen until the dose of methylprednisolone had been tapered to 32 mg/day to avoid infections. None of the patients developed any severe infections, hypogammaglobulinemia, or leukopenia, and any side effects that required treatment discontinuation were observed in this regimen.

The combined use of CYC and RTX can enables the administration of lower cumulative doses of CYC. As a result, we concluded that the combined therapy is necessary to minimize the risks such as secondary malignancy and infertility. 

#### 4.2.2. Maintenance therapy

Joode et al. [26] found that the use of AZA within less than 12 months of maintenance therapy was associated with increased risk of relapse. In our study, the duration of AZA treatment was 24 months in patients with and without relapse. Thus, AZA use was not found to be a risk factor for relapse.

In the WEGENT trial that compared MTX and AZA, similar relapse rates were obtained (54% vs. 60%) [27]. Our study also found no difference between AZA and MTX in terms of relapse rates.

In the MAINRITSAN trial that compared RTX and AZA in a maintenance therapy, the relapse rates were lower in the RTX group [28]. By contrast, our study found no difference between RTX and AZA in terms of relapses.

Besada et al. [23] reported that the use of RTX in maintenance therapy led to a lower rate of relapse; however, treatment cessation was needed in one-third of the patients due to infections. In our series, only two patients stopped the RTX use due to side effects during the maintenance therapy. RTX was discontinued because of hepatitis B activation.

Furthermore, Roderau et al. [7] found that mortality rates were higher in the group that suffered relapses (19% vs. 2%, P = 0.0084). Our study similarly found that mortality rates were higher in the group that had relapses (30% vs. 7.7%, P = 0.06). The overall mortality rate in our study was 18%. Although the survival period was longer in patients who did not suffer relapses, the difference was not significant. As regards relapse and survival, Roderau et al. [7] reported that survival was shorter in the group with relapses, but the difference was not significant, consistent with our results.

Although relapses are common in the clinical course of GPA, survival rates have been gradually improving, which may have stemmed from the new treatment regimens, from closer follow-ups of patients, and from earlier detection of relapses. 

Our study has a limitation, which is that it was a single-center, retrospective study involving a limited number of patients. Prospective multicenter studies are warranted to identify the risk factors affecting recurrence rates in GPA patients. However, one strength of our study is that it involved a homogeneous patient population consisting of GPA patients only. Another strength is that the patients were regularly followed-up by the same physician. Moreover, our study had longer follow-up period than the other studies.

## 5. Conclusion

Determining the correlation between the clinical signs at the time of diagnosis and relapses in GPA patients is important in order to determine the prognosis and mortality risks of these patients. Our study showed that patients with cavitary lung lesions and high serum creatinine levels (>1.15 mg/dL) display an increased risk for relapse. We conclude that patients with these risk factors should be monitored carefully and closely for relapse. In patients with severe organ involvement in GPA, the combined use of CYC and RTX may reduce relapse, mortality, and morbidity rates. 

## Informed consent

This study was approved by the Başkent University Institutional Review Board (project no.: KA19/287) and was supported by the Başkent University Research Fund.

## References

[ref1] (2014). Granulomatosis with polyangiitis (Wegener): clinical aspects and treatment. Autoimmunity Reviews.

[ref2] (2018). The complications of vasculitis and its treatment. Best Practice & Research: Clinical Rheumatology.

[ref3] (2008). Outcomes from studies of antineutrophil cytoplasm antibody associated vasculitis: a systematic review by the European league against rheumatism systemic vasculitis task force. Annals of the Rheumatic Diseases.

[ref4] (2008). Predictors of treatment resistance and relapse in antineutrophil cytoplasmic antibody-associated small-vessel vasculitis: comparison of two independent cohorts. Arthritis & Rheumatism.

[ref5] (2019). Treatment strategies in ANCA-associated vasculitis. Current Rheumatology Reports.

[ref6] (2017). Clinical characteristics and outcome of Spanish patients with ANCA-associated vasculitides: impact of the vasculitis type, ANCA specificity, and treatment on mortality and morbidity. Medicine (Baltimore).

[ref7] (2020). Relapses in patients with anti-neutrophil cytoplasmic antibody-associated vasculitis: a retrospective study. Clinical Rheumatology.

[ref8] (2005). Predictors of relapse and treatment resistance in antineutrophil cytoplasmic antibody-associated small-vessel vasculitis. Annals of Internal Medicine.

[ref9] (1992). Wegener granulomatosis: an analysis of 158 patients. Annals of Internal Medicine.

[ref10] (2018). Prognostic factors for survival and relapse in ANCA-associated vasculitis with renal involvement: a clinical long-term follow-up study. International Journal of Nephrology.

[ref11] (2020). Spectrum and prognosis of antineutrophil cytoplasmic antibody-associated vasculitis-related bronchiectasis: data from 61 patients. The Journal of Rheumatology.

[ref12] (2018). Prognostic significance of cavitary lung nodules in granulomatosis with polyangiitis (Wegener’s): a clinical imaging study of 225 patients.

[ref13] (2003). Active disease and residual damage in treated Wegener’s granulomatosis: an observational study using pulmonary high-resolution computed tomography. European Radiology.

[ref14] (2008). Relapses in patients with antineutrophil cytoplasmic autoantibody-associated vasculitis: likely to begin with the same organ as initial onset. The Journal of Rheumatology.

[ref15] (2012). Risk factors for relapse of antineutrophil cytoplasmic antibody-associated vasculitis. Arthritis & Rheumatology.

[ref16] (2014). Predictors of treatment resistance and relapse in antineutrophil cytoplasmic antibody-associated vasculitis: a study of 439 cases in a single Chinese center. Arthritis & Rheumatology.

[ref17] (2018). The utility of urinalysis in determining the risk of renal relapse in ANCA-associated casculitis. Clinical Journal of the American Society of Nephrology.

[ref18] (2014). Characteristics, prognosis, and outcomes of cutaneous ischemia and gangrene in systemic necrotizing vasculitides: a retrospective multicenter study. Seminars in Arthritis and Rheumatism.

[ref19] (2012). Value of ANCA measurements during remission to predict a relapse of ANCA-associated vasculitis — a meta-analysis. Rheumatology (Oxford).

[ref20] (2010). Rituximab versus cyclophosphamide for ANCA-associated vasculitis. The New England Journal of Medicine.

[ref21] (2020). Treatment and its side effects in ANCA-associated vasculitides - study based on POLVAS registry data. Advances in Medical Sciences.

[ref22] (2015). Rituximab versus cyclophosphamide in ANCA-associated renal vasculitis: 2-year results of a randomised trial. Annals of the Rheumatic Diseases.

[ref23] (2013). Long-term efficacy and safety of pre-emptive maintenance therapy with rituximab in granulomatosis with polyangiitis: results from a single centre. Rheumatology.

[ref24] (2017). Combination therapy with rituximab and cyclophosphamide for remission induction in ANCA Vasculitis. Kidney International Reports.

[ref25] (2019). A novel glucocorticoid-free maintenance regimen for anti-neutrophil cytoplasm antibody-associated vasculitis. Rheumatology.

[ref26] (2017). et al. Long term azathioprine maintenance therapy in ANCA-associated vasculitis: combined results of long-term follow-up data. Rheumatology (Oxford).

[ref27] (2016). French vasculitis study group. Long-term outcomes among participants in the WEGENT trial of remission-maintenance therapy for granulomatosis with polyangiitis (Wegener’s) or microscopic polyangiitis. Arthritis & Rheumatology.

[ref28] (2018). French vasculitis study group. Long-term efficacy of remission-maintenance regimens for ANCA-associated vasculitides. Annals of the Rheumatic Diseases.

